# Engineering the 3′‐UTR of Tobacco Vein Mottling Virus to Confer Cross‐Protection Against Potyviruses

**DOI:** 10.1111/mpp.70268

**Published:** 2026-05-01

**Authors:** Haijuan Wang, Zhenqi Sun, Baolong Zhang, Zhaoran Wu, Jiaxin Xu, Mingmin Zhao

**Affiliations:** ^1^ College of Horticulture and Plant Protection Inner Mongolia Agricultural University Hohhot China

**Keywords:** 3′‐UTR, cross protection, *Tobacco vein mottling virus*, viral infection symptom, viral infectivity

## Abstract

Tobacco vein mosaic virus (TVMV) is a member of the family *Potyviridae*. We explored if engineering the 3′ noncoding RNA would alter viral infection symptoms and viral infectivity of TVMVwt, which could be used for cross‐protection. We engineered an infectious clone of TVMV mutant virus pLX‐TVMV58 (TVMV58), which contains a 58‐nt 'AT' fragment in the 3′‐untranslated region (UTR) of the full‐length cDNA infectious clone pLX‐TVMVwt (TVMVwt), using a one‐step assembly method. We explored whether the mechanism of the attenuated symptoms in *Nicotiana benthamiana* caused by TVMV58 compared with that by TVMVwt is linked to the stem structure of TVMV58 3′‐UTR. The viral infectivity of the mutant virus TVMV58mutA (with three loops in the structure of TVMV58 3′‐UTR) was slightly higher than TVMV58, whereas the viral infectivity of the mutant virus TVMV58mutB (with two loops in the structure of TVMV58 3′‐UTR) decreased significantly. However, we observed that the mutant virus TVMV58mutC (with no loop in the structure of TVMV58 3′‐UTR) or TVMVmutTG lost infectivity. The infectivity of TVMV58 and its mutants was independent of the RNA silencing mechanism, which was supported by a similar infection pattern in *NbDCL*‐silenced plants. Moreover, TVMV58 showed high cross‐protection against PVY‐Ros1 (potato virus Y isolate carrying the *Rosea1* gene) and TuMV‐GFP (turnip mosaic virus expressing GFP) in *N. benthamiana*, but showed no cross‐protection effect against tobacco mosaic virus (TMV) infection.

## Graphical abstract

The 3′‐untranslated region (3′‐UTR) and its secondary structure of virus genomic RNA of tobacco vein mottling virus (TVMV) are important in symptoms attenuation, which can be engineered for cross‐protection against further viral infection. We provide experimental evidence that supports the roles of 3′‐UTR in potyvirus infection.

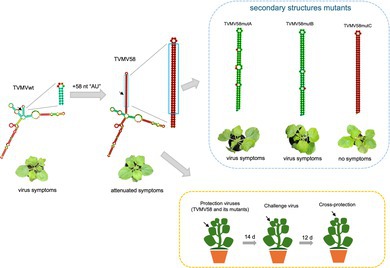

## 1 Introduction

Tobacco vein mottling virus (TVMV) was reported in the southeastern United States on tobacco (*Nicotiana tabacum* 'Burley') (Gooding and Rufty 1987). It subsequently occurred in tobacco‐growing areas of Kentucky, Tennessee and Virginia (Brantley and Hunt 1993; Maiti et al. 1993; Atreya et al. 1995; Blanc et al. 1997). TVMV is widely distributed in perennial solanaceous plants and is transmitted with high efficiency by several aphid species in a non‐persistent manner. Typically, TVMV causes phenotypic changes including vein banding, leaf discolouration, curling and dwarfing.

*Tobacco vein mottling virus* is a member of the *Potyviridae* family, which includes a large group of plant RNA viruses with single‐stranded (+)‐sense RNA with an average size of ~9.7 kb (Sun et al. 2010). In addition to being 3′‐polyadenylated and containing a 5′‐terminal genome‐linked protein (VPg), TVMV RNA encodes 11 putative proteins: protein (P1), helper component‐proteinase (Hc‐Pro), protein 3 (P3), N‐terminal half of P3 fused to PIPO (P3N‐PIPO), 6 kDa kinase 1 (6K1), cylindrical inclusion (CI), 6 kDa kinase 2 (6K2), viral genome‐linked protein (VPg), nuclear inclusion a proteinase (NIa‐Pro), nuclear inclusion b (NIb) and coat protein (CP) (Domier et al. 1986).

The 253‐nucleotide (nt) long TVMV 3′‐untranslated region (UTR) is highly structured. However, the role of the 3′‐UTR in TVMV infection remains unknown. Studies have shown that a determinant of disease symptom severity was located in the 3′‐terminal noncoding region in TVMV (Rodríguez‐Cerezo et al. 1991). They discovered that inoculation of *N. tabacum* with RNA transcribed in vitro from a variant (pXBS8) of the cloned full‐length cDNA of TVMV resulted in the attenuation of vein mottling and blotching symptoms typically caused by transcripts of cloned wild‐type cDNA (pXBS7). Comparing with the sequence of pXBS7, a 58nt AU‐rich fragment (four adjacent direct repeats of a 14‐nt sequence, AUAAUUAUAUAUAU, and two additional nucleotides (AU) between the first and second repeats) was present in the 3′‐UTR of TVMV RNA (Rodríguez‐Cerezo et al. 1991). These results indicate that a noncoding region of the genome can have a direct effect on the induction of disease symptoms by mutant TVMV. However, it is not clear why attenuated symptoms are induced by the insertion of this noncoding sequence into the TVMV genome.

Construction of an infectious clone is a universal approach applied in disease causality studies and reverse genetics of viruses to facilitate the study of virus biology (Pasin et al. 2019; Zhao et al. 2020). The first successful construction of an infectious clone was that of *Brome mosaic virus*, a plant RNA virus (Ahlquist et al. 1984). With the continuous efforts of plant virologists, efficient construction of infectious clones of various plant RNA viruses has been achieved, laying a technical foundation for studying viral pathogenesis and disease resistance (Niu et al. 2014; Shi et al. 2019). We obtained an infectious clone of TVMV (pLX‐TVMVwt) by one‐step Gibson assembly technology with a custom enzymatic premix and confirmed by Illumina sequencing (Zhao et al. 2020). In this study, we designed a construction strategy to transfer the viral sequence of TVMVpXBS8 into a binary vector to facilitate an agroinfiltration assay. Similarly, the 58‐nt ‘AT’‐rich repeat sequence was cloned into pLXB‐TVMV to create the mutant viral construct pLX‐TVMVpXBS8 (TVMV58).

Studies have shown that noncoding RNAs are involved in regulating key biological processes in plants at multiple levels, including reproductive development, environmental adaptation and stress resistance. This multidimensional regulatory characteristic reveals the complexity of the life system and provides an innovative direction for analysing life regulatory networks and developing new biotechnology tools (Bazzini et al. 2007). In recent years, the function of viral noncoding RNAs in regulating host–pathogen interactions and symptom production has become a growing area of interest in the field of virology. tRNA‐like secondary structures in the 3′ noncoding region of plant virus mRNAs may also be involved in symptom formation. For example, tRNA‐like secondary structure mutations in the 3′ noncoding region of tobacco mosaic virus (TMV), such as inserting a longer poly(A) sequence upstream of pseudoknots, can reduce viral accumulation and induce milder symptoms in tobacco (Guo et al. 2015). Deep sequencing technology showed that TMV mutants containing a poly(A) sequence had reduced defence responses in tobacco (Guo and Wong 2017). Additionally, an AU‐rich hairpin structure in the genome of tomato spotted wilt virus (TSWV) can induce RNA silencing in the host (Hedil et al. 2014). The important secondary structures in the 3′ noncoding region of pepino mosaic virus (PepMV) RNA were identified using partially purified viral RNA polymerase. An RNA pseudoknot can form in the 3′‐UTR, containing part of the poly(A) tail. Transfection of tobacco mesophyll protoplasts using PepMV RNA with only twoA residues (pA2) in the tail resulted in almost undetectable accumulation of gRNA and sgRNA. However, replication was significantly enhanced by the presence of six A residues (pA6), proving that the pseudoknot is required for PepMV RNA replication (Olsthoorn et al. 2022).

Recent studies indicated that disruption of the CP gene or the stem‐loop (SL)‐like structure in the 3′‐UTR of TMV can enhance the replication ability of the virus (Gao et al. 2012). The function of the 3′‐UTR of potyviruses has not been fully elucidated. A mutant of clover yellow vein virus (ClYVV) with poly(A) deletion at the 3′‐terminal was repaired during the infection of plants (Tacahashi and Uyeda 1999). In another study, ClYVV mutants lacking a different region of the 3′‐UTR were unable to infect a broad bean host (Sekiguchi et al. 2003).

In this study, mutant viruses of TVMV58, including a 58 nt AU fragment in the 3′‐UTR region, were designed based on the infectious clone of pLX‐TVMV58 to address whether secondary structures or certain nucleotides in the 3′‐UTR region of TVMV58 are associated with attenuated symptoms and viral infection. Viral infectious clones have been widely used in many fields, such as gene functions of viruses, pathogenesis, application of expression vectors, virus‐induced gene silencing for gene function verification and cross‐protection of weak strains. We also tested the cross‐protective effects of TVMV58 against turnip mosaic virus (TuMV), potato virus Y (PVY) and TMV.

## 2 Results

### 2.1 One‐Step Assembly of a Full‐Length cDNA Infectious Clone of TVMV58

**FIGURE 1:**
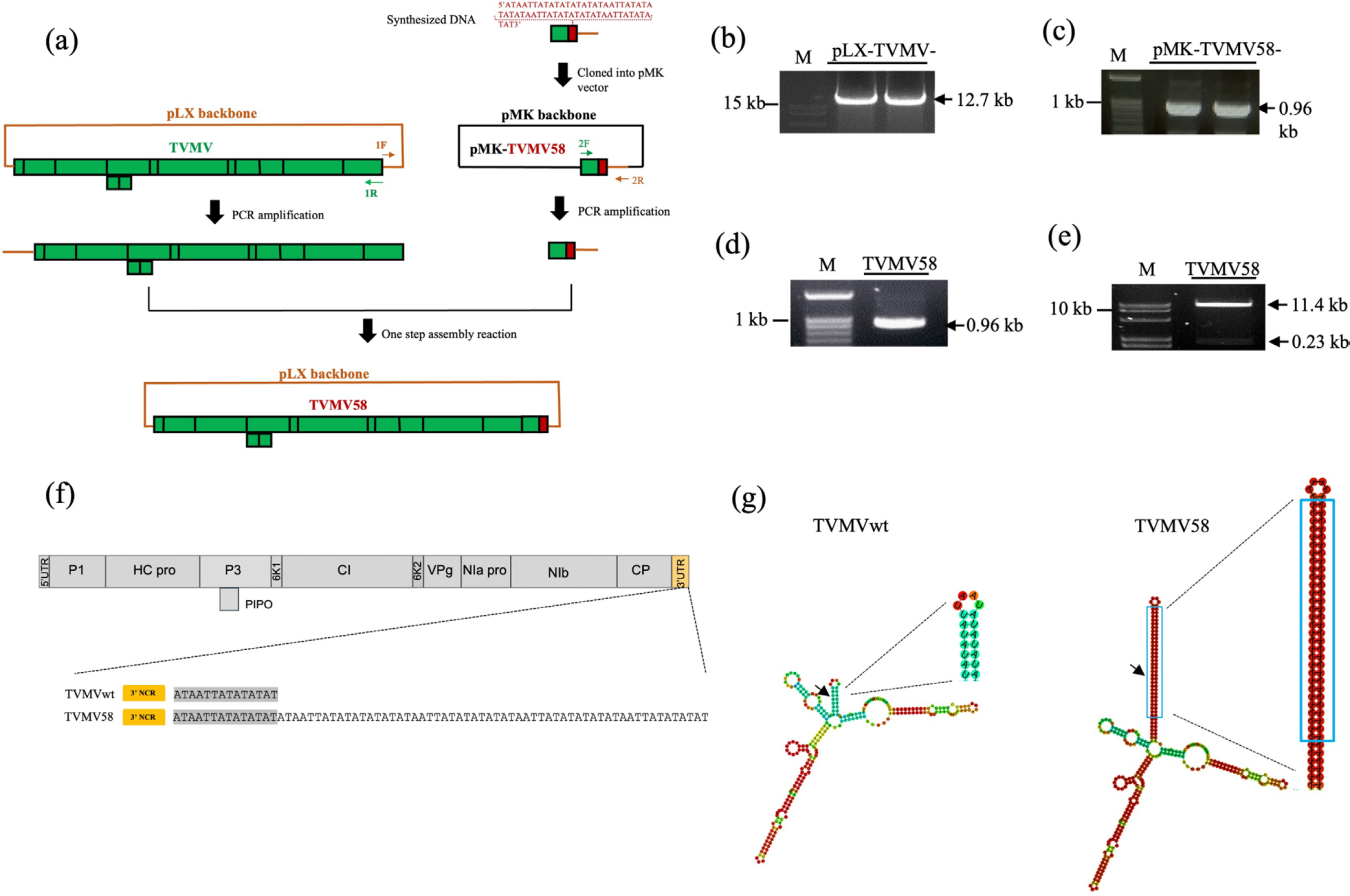
One‐step Gibson assembly of a full‐length cDNA infectious clone of TVMV58. (a) The diagram design of pLX‐TVMV58 (TVMV58) cloning. A 963‐nt DNA fragment including partial 3′‐untranslated region (UTR) sequence of pLX‐TVMVwt, 58‐nt 'AT' and partial sequence of pLX vector was synthesized and cloned into pMK vector to obtain pMK‐TVMV58. Primer 2F/2R was used to amplify a fragment. Using the infectious clone of pLX‐TVMVwt (TVMVwt) as the template, a fragment including TVMVwt sequences and pLX backbone was amplified by primer 1F/1R. Two cDNA fragments were assembled by one‐step Gibson assembly reaction to generate pLX‐TVMV58. The primers used are indicated (green and orange arrows). (b, c) Fragments obtained by PCR with primer 1F/1R and 2F/2R in agarose gel. (d) Confirmation of TVMV58 after transformation into *Escherichia coli* DH5α by PCR amplification with primer 2F/2R. A 0.96 kb fragment was obtained. (e) Digestion profiles of purified plasmids from selected clones. DNA sizes of marker are shown on the right. The size of XbaI‐digestion fragments is shown on the right. (f) The viral genome of TVMV and the partial sequences of 3′ UTR RNA of TVMVwt and TVMV58 are shown. (g) The secondary structure of 3′‐UTR in TVMVwt and TVMV58 mutants predicted by RNAFold. The difference stem of TVMVwt and TVMV58 was shown.

Previous studies showed that pLX‐TVMVpXBS8, a variant strain of TVMV generated during infection by the wild‐type virus under natural conditions, contained a 58 nt fragment inserted at positions 9239–9252 of the 3′‐UTR compared with the wild‐type TVMV virus (Rodríguez‐Cerezo et al. 1991). Based on the infectious clone of pLX‐TVMVwt described previously (Zhao et al. 2020), a DNA fragment (968 nt) including a partial 3′‐UTR sequence of pLX‐TVMVwt, a 58‐nt 'AT' fragment, and a partial sequence of the pLX vector was synthesized and cloned into the pMK vector using GeneArt gene synthesis (Thermo Fisher Scientific) to obtain pMK‐TVMV58 (Figure 1a, Figure S1a). Primer 2F/2R was used to amplify the synthesized fragments (Figure 1c). Using an infectious clone of pLX‐TVMVwt (TVMVwt) as the template, the TVMVwt viral sequence and pLX backbone, including the cauliflower mosaic virus (CaMV) 35S promoter and nopaline synthase (nos) terminator sequences, were amplified using primers 1F/1R (Figure 1b). Primers 1F/1R and 2F/2R were designed to generate DNA fragments with overlapping termini compatible with the Gibson assembly (Table S1).

Using a custom premix, two cDNA fragments (12.7 kb and 0.96 kb) were cloned into pLX‐TVMV58 by Gibson assembly. The preparation of the custom enzymatic premix for the one‐step Gibson assembly has been previously described (Zhao et al. 2020). The plasmids were confirmed using PCR and restriction analysis by XbaI digestion (Figure 1d,e). DNA sequencing results showed that the pLX‐TVMV58 plasmid contained an insertion of a 58‐nt 'AT' sequence (Figure S1b). These results confirmed the successful engineering of a mutant viral infectious clone of TVMV58. The 3′‐UTR of TVMV58 was compared with that of the TVMVwt (Figure 1f). Secondary structure prediction of the partial sequence (332 nt) showed that a long stem appeared in TVMV58 compared to that in TVMVwt (Figure 1g).

### 2.2 TVMV58 Caused the Attenuated Symptoms in *Nicotiana benthamiana*

**FIGURE 2:**
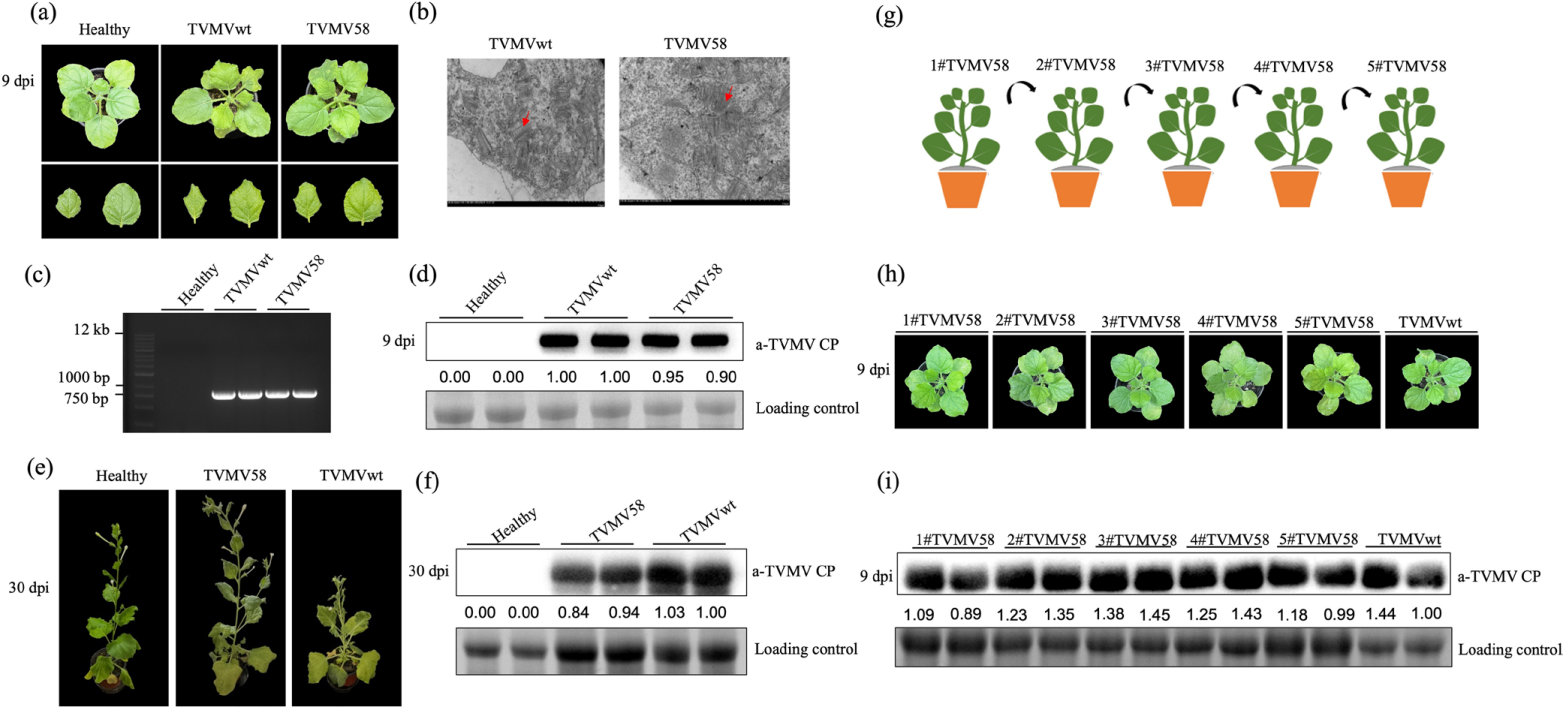
Agro‐infiltration of pLX‐TVMV58 infection clone in *Nicotiana benthamiana*. (a) Infectious clones of pLX‐TVMVwt(TVMVwt) and pLX‐TVMV58(TVMV58) were transformed into *Agrobacterium tumefaciens* and delivered to *N. benthamiana* plants by agro‐infiltration. Symptoms of TVMVwt, TVMV58 and healthy plants were photographed at 9 days post‐infiltration (dpi). (b) Transmission electron micrograph of particles and pinwheel‐like inclusions observed in *N. benthamiana* plants infected with TVMVwt and TVMV58; scale bar, 200 nm. Red arrow shows the viral particles. (c) Viral accumulation was detected by reverse transcription‐PCR. (d, f) The viral accumulation of TVMV upper leaves was detected by western blot with anti‐CP of TVMV. Healthy plant without any treatment used as the control; bands of RuBisCO are shown as a loading control. (e) Symptoms of the plants treated with TVMVwt, TVMV58 and healthy plants at 30 dpi. (g) The schematic shows passage infection of TVMV58 by manual inoculation in *N. benthamiana* plants. The upper leaves were collected and used as the inoculum for serial passage infection. (h) Symptoms of passage infection plants were observed and photographed at 9 days post‐inoculation. A plant infected with TVMV wild‐type virus is shown as a control. (i) Viral accumulation was detected by anti‐TVMV CP immunoblotting of upper uninoculated leaves; Bands of RuBisCO are shown as a loading control. The CP signal in each band in western blot was quantified by ImageJ.

The plasmid pLX‐TVMV58 was transformed into *Agrobacterium tumefaciens* C58C1 and delivered to *N. benthamiana* plants using an agroinfiltration assay to test the infectivity of TVMV58. The infectious clone pLX‐TVMVwt (TVMVwt) was used as a control. The results showed that the typical vein banding symptoms were observed in plants infiltrated with TVMVwt at 9 days post‐infiltration (dpi), whereas plants treated with TVMV58 showed obviously attenuated symptoms (Figure 2a).

Viral particles and pinwheel‐like inclusions were observed in *N. benthamiana* plants infected with TVMVwt or TVMV58 using transmission electron microscopy (Figure 2b). Filamentous virions were observed, and there was no significant difference between TVMVwt‐ and TVMV58‐infected plants. Similar levels of viral accumulation in TVMVwt and TVMV58 infected plants were detected by reverse transcription (RT)‐PCR (Figure 2c) and western blotting with anti‐CP antibodies against TVMV (Figure 2d). When the treated plants were allowed to grow for 30 days, we observed that the TVMVwt plants showed evident symptoms of dwarfing and leaf shrinkage. However, TVMV58 plants showed attenuated symptoms that were similar to those of healthy plants (Figure 2e). After 30 days, the viral accumulation in TVMV58 was slightly decreased compared to TVMVwt (*p* = 0.19) (Figure 2f).

Crude extracts from plants agro‐inoculated with pLX‐TVMV58 were used as the inoculum for serial infection of healthy plants to verify whether the engineered mutant virus TVMV58 could be maintained after several passages in *N. benthamiana* (Figure 2g). Plants infected with the TVMVwt virus were used as the control. The symptoms of plants from passages 2#–5# of TVMV58 were similar to those of the original 1#TVMV58 plants at 9 dpi (Figure 2h). Viral accumulation in the plants was detected using anti‐TVMV CP immunoblotting of upper uninoculated leaves, and there was no significant difference between five successive passages of TVMV58 (1#–5#) and the TVMVwt control. This indicated that the stability of TVMV58 infection was maintained up to the 5th passage (Figure 2i). RT‐PCR and sequencing were used to detect the 58 nt fragment in TVMV58 (1#–5#) (Figure S2a,b). This indicated that the infectious clone of TVMV58 efficiently infected *N. benthamiana* plants and caused attenuated symptoms.

### 2.3 The Structure of the 58‐nt 'AU' Fragment Is Crucial for Viral Infection

**FIGURE 3:**
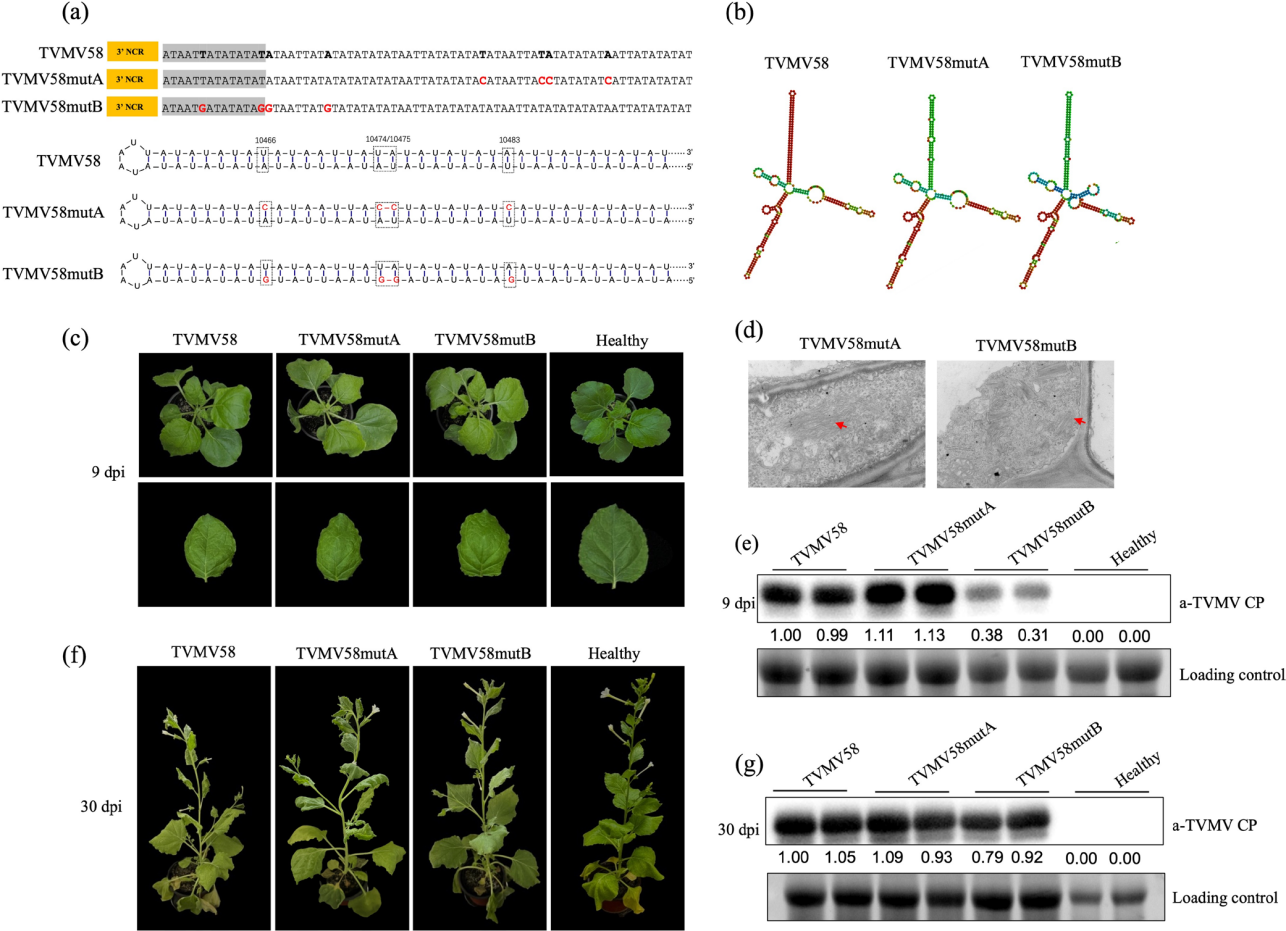
The design of TVMV58 mutant viruses and infection in *Nicotiana benthamiana*. (a) The partial sequences of 3′ untranslated region (UTR) in TVMV58 and TVMV58mutA and TVMV58mutB are shown. The position in TVMV58 used to modify for creating mutations is shown in black bold font. The mutated nucleotides in TVMV58mutA and TVMV58mutB are shown in red font. To introduce the three loops, the nucleotides with square and red colour at the 3′ end of the stem of 3′‐UTR in TVMV58 were mutated at the corresponding position in TVMV58 sequence, which created the TVMV58mutA. In the case of TVMV58mutB, mutations were designed to obtain two loops by modifying the 5′ end sequences of the stem of 3′‐UTR in TVMV58. (b) The secondary structure of 3′‐UTR in TVMV58 and TVMV58 mutants predicted by the RNAFold is shown. The loops are indicated. (c) The infectious clones of TVMV58, TVMV58mutA and TVMV58mutB were agro‐infiltrated in *N. benthamiana*. Infection symptoms were photographed at 9 days post‐infiltration (dpi). Healthy plant without any treatment was used as the control. (d) Transmission electron micrograph of particles observed in *N. benthamiana* infected with TVMV58 and mutant viruses; scale bar, 200 nm. (e) Viral accumulation in upper uninoculated *N. benthamiana* leaves at 9 dpi was assessed by western blot with anti‐TVMV CP; healthy plant without any treatment was used as the control; RuBisCO protein is shown as a loading control. (f) Symptoms of *N. benthamiana* plants infected with TVMV58, TVMV58mutA and TVMV58mutB were photographed at 30 dpi. (g) The viral accumulation level in plants infected with TVMV58, TVMV58mutA and TVMV58mutB mutant viruses was detected by western blot with anti‐TVMV CP. The CP signal in each band in the western blot was quantified by ImageJ.

The 58‐nt 'AU' fragment is a determinant of attenuated symptoms of TVMV infection in vitro (Rodríguez‐Cerezo et al. 1991). In order to explore the importance of the 58‐nt 'AU' fragment in TVMV58, the stem structure of TVMV58 was predicted (Figure 1g). We propose a hypothesis that either this sequence of the 58‐nt 'AU' fragment or the secondary structure of the 3′‐UTR processed during viral replication is crucial for the attenuated symptoms in plants infected with TVMV58. Therefore, a mutant virus of TVMV58mutA with four nucleotides modified from ‘U to C’ or ‘A to C’ in the positions 10,466, 10,474, 10,475 and 10,483 of infectious clone pLX‐TVMV58 was created based on the secondary structure of the 3′‐UTR in TVMV58 (Figure 3a, Figures S3 and S4). Compared to the predicted secondary structure of TVMV58, three loops in the stem region are predicted in TVMV58mutA, wherease in TVMV58mutB four nucleotides are modified from ‘A to G’ or ‘U to G’ in the same positions to generate a secondary structure with two loops in the stem region (Figure 3b, Figure S3).

Infectious clones of TVMV58, TVMV58mutA and TVMV58mutB were transformed into *A. tumefaciens* C58C1 and infiltrated into *N. benthamiana* (Figure S4). At 9 dpi, the typical symptoms of leaf deformation and vein banding were observed in plants infected with TVMVwt, TVMV58, TVMV58mutA or TVMV58mutB (Figure 3c). No differences in viral particles were observed between TVMV58mutA and TVMV58mutB (Figure 3d), as well as between TVMVwt and TVMV58 (Figure 2b). Viral accumulation in plants treated with TVMV58mutA increased slightly, whereas a dramatic decrease was detected in plants treated with TVMV58mutB (Figure 3e). Symptoms of viral infection were observed at 30 dpi. The symptoms of TVMV58mutA‐infected plants were similar to those of TVMV58‐infected plants, but the symptoms caused by TVMV58mutB were slightly milder (Figure 3f). Viral accumulation of TVMV58, TVMVmutA and TVMVmutB was not significantly difference at 30 dpi (Figure 3g).

### 2.4 Two Nucleotides in the 3′‐UTR of TVMVwt are Essential for Viral Infection

**FIGURE 4:**
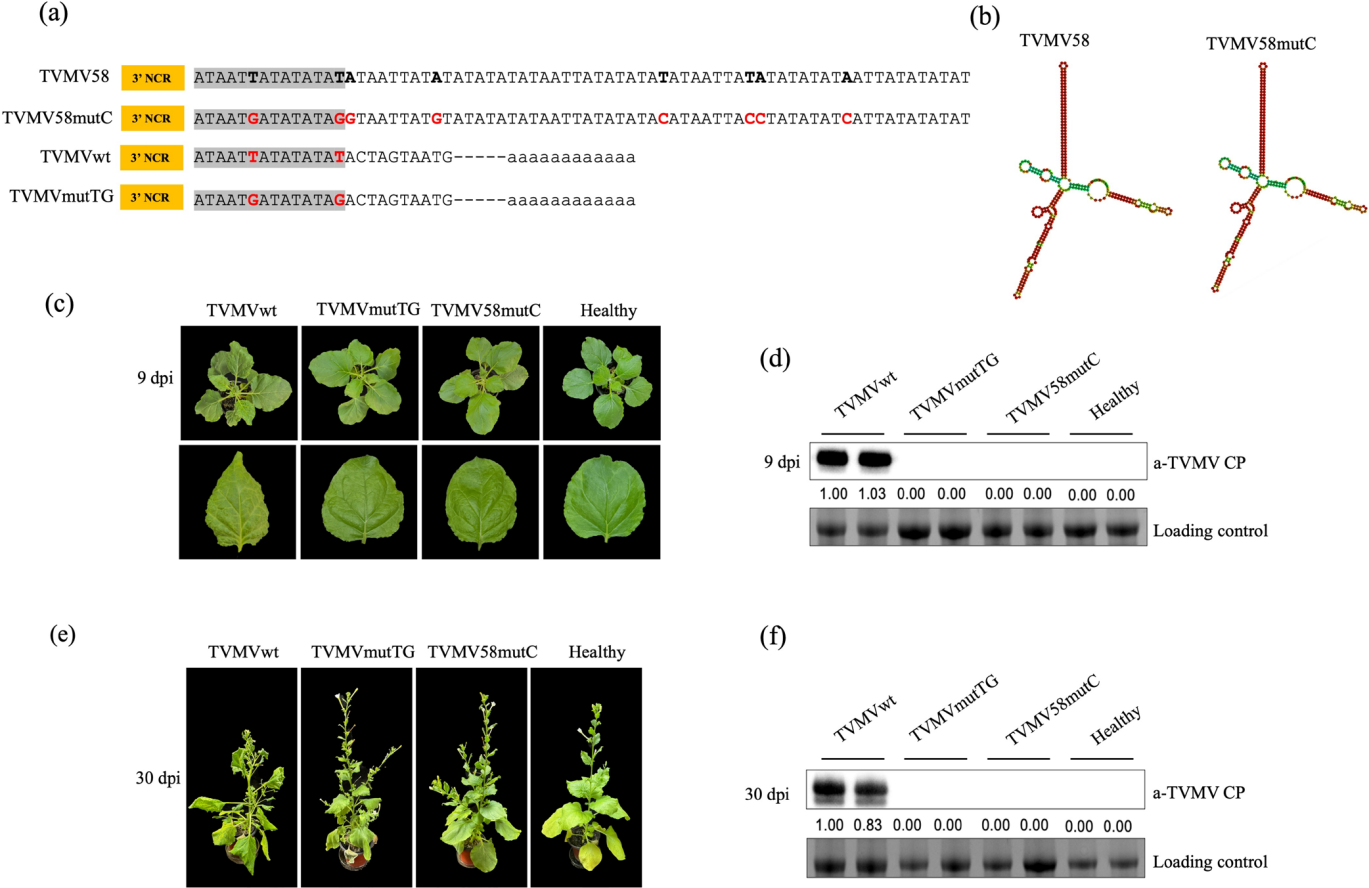
The complementary stem structure of TVMV58 and two point mutations in TVMVwt lose viral infection activity. (a) The partial sequences of the 3′ untranslated region (UTR) in TVMV58mutC and TVMV58mutTG are shown. The 8 nucleotide mutations in TVMV58 shown in red font were modified to obtain TVMV58mutC. Mutation from T to G (in red font) in the 3′‐UTR of TVMVwt was used to obtain TVMVmutTG. (b) The secondary structure of the 3′‐UTR in TVMV58mutC and TVMV58mutTG predicted by RNAFold is shown. The stem structures are shown. (c) The infectious clones of TVMVwt, TVMVmutTG and TVMV58mut viruses were agro‐infiltrated in *Nicotiana benthamiana*. Infection symptoms were photographed at 9 days post‐infiltration (dpi). A healthy plant without any treatment was used as the control. (d) Viral accumulation in upper uninoculated *N. benthamiana* leaves at 9 dpi was assessed by western blot with anti‐TVMV CP; healthy plant without any treatment was used as the control; RuBisCO protein is shown as a loading control. (e) Symptoms of *N. benthamiana* plants infected with TVMVwt, TVMVmutTG and TVMV58mutC were photographed at 30 dpi. (f) The viral accumulation level in the above plants was detected by western blot with anti‐TVMV CP. The CP signal in each band in the western blot was quantified by ImageJ.

We designed a TVMV58mutC mutant, including mutations at positions 10,428 (T/G), 10,436 (T/G), 10,437 (A/G), 10,445 (A/G), 10,466 (T/C), 10,474 (T/C), 10,475 (A/C) and 10,483 (A/C) of TVMV58 (Figure 4a, Figure S5), which had the same stem structure as TVMV58 (Figure 4b) to explore if we could restore the infectivity of TVMV58mutB by closing the loops of the TVMV58mutB structure. Because of the presence of two nucleotides at positions 10,428 (T/G) and 10,436 (T/G) in TVMV58 and TVMV58mutC, we considered that this might also be a determinant for viral infection or attenuation of symptoms. We designed a mutant infectious clone named TVMV58mutTG by modifying the nucleotides T to G at positions 10,428 (T/G) and 10,436 (T/G) (Figure 4a, Figure S6).

Infectious clones of TVMV58mutC and TVMV58mutTG were used to infiltrate *N. benthamiana*. At 9 dpi, we observed that plants infiltrated with TVMV58mutC and TVMV58mutTG showed no symptoms of viral infection, whereas typical vein mottling symptoms were observed in plants infiltrated with TVMVwt (Figure 4c, Figure S7). Correspondingly, no viral accumulation was detected in the TVMV58mutC and TVMV58mutTG samples (Figure 4d). The growth status of TVMV58mutC and TVMVmutTG was consistent with that of healthy plants at 30 dpi; no typical symptoms of TVMVwt were observed, and no viral accumulation was detected (Figure 4e,f).

### 2.5 The Infection of the TVMV58 and TVMV58 Mutants Does Not Depend on the DCL Homologues

**FIGURE 5:**
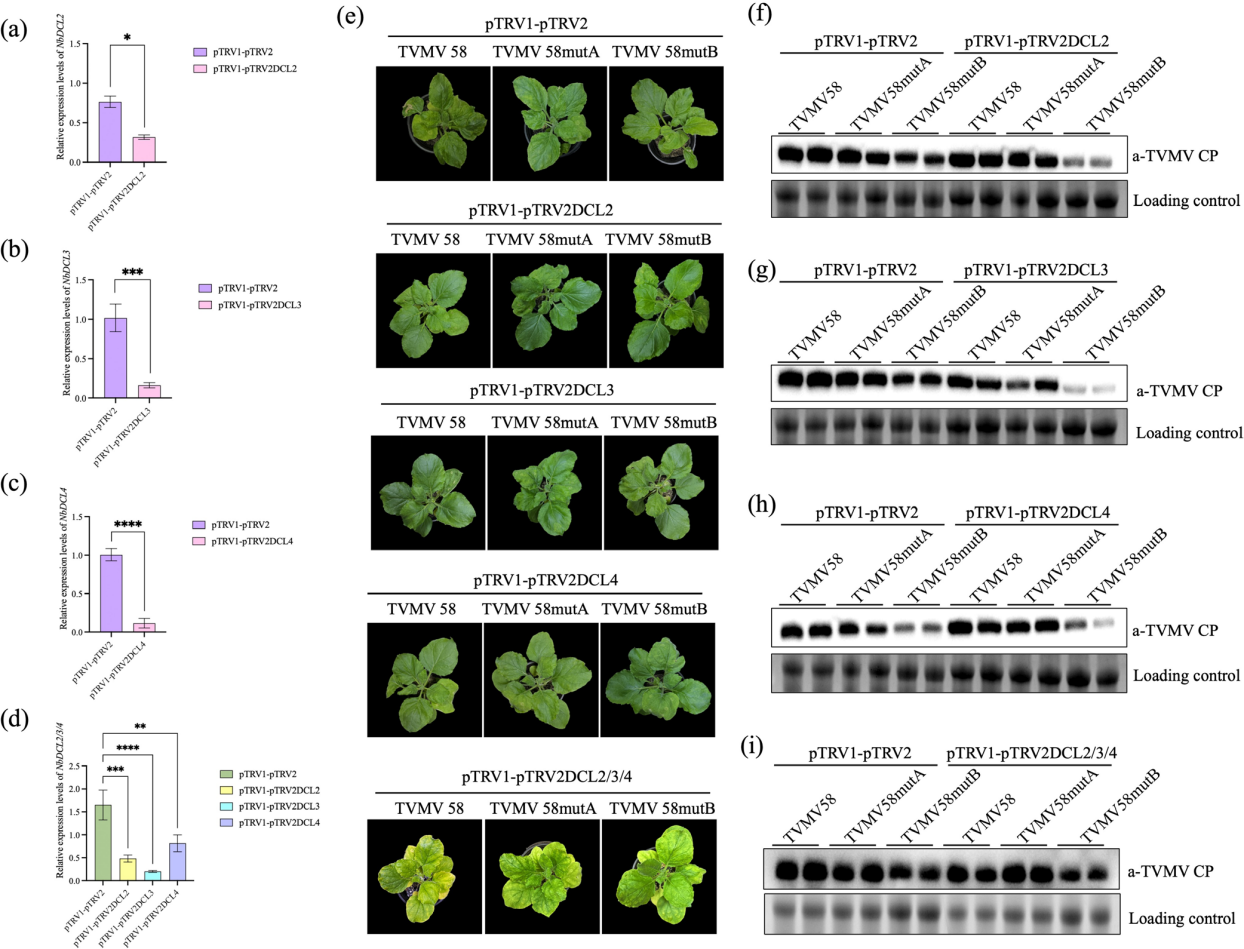
Silencing *NbDCL2*, *NbDCL3* and *NbDCL4* by TRV and detection of effect on plants. (a) *NbDCL2* silencing effect. (b) *NbDCL3* silencing effect. (c) *NbDCL4* silencing effect. (d) *NbDCL2*, *NbDCL3* and *NbDCL4* co‐silencing effect. (e) Symptoms of plants following silencing by infiltration with pTRV constructs, then mechanical inoculation with TVMV58, TVMV58mutA, or TVMV58mutB. Silencing constructs: pTRV1+pTRV2 empty vector. pTRV1+pTRV2‐NbDCL2, pTRV1+pTRV2‐NbDCL3 pTRV1+pTRV2‐NbDCL4. (f) Detection by western blot of viral accumulation of TVMV58, TVMV58mutA and TVMV58mutB in *NbDCL2* silenced plants. (g) Western blot was used to detect virus accumulation of TVMV58, TVMV58mutA and TVMV58mutB in *NbDCL3* silenced plants. (h) Western blot was used to detect virus accumulation of TVMV58, TVMV58mutA and TVMV58mutB in *NbDCL4* silenced plants. (i) Western blot was used to detect virus accumulation of TVMV58, TVMV58mutA and TVMV58mutB in *NbDCL2*, *NbDCL3* and *NbDCL4* co‐silenced plants. The antibody is anti‐TVMV CP, and the loading control is a RuBisCO protein.

DCLs produce small interfering RNA (siRNAs) and microRNAs as essential components of RNA silencing. We created TRV‐based silencing vectors to silence *DCL* homologues in *N. benthamiana* to investigate whether the infectivity changes in TVMV58 and TVMV58 mutants were linked to the cleavage by DCL during RNA silencing. The expression level of *DCL* was significantly decreased in *N. benthamiana* plants infiltrated with silencing vectors of *NbDCL2*, *NbDCL3* or *NbDCL4* at 14 dpi (Figure 5a–c; Figure S8). Individually silencing *NbDCL2*, *NbDCL3* or *NbDCL4* did not alter the viral symptoms or viral infectivity of TVMV58, TVMV58mutA or TVMV58mutB after inoculation (Figure 5e) compared to the results shown in Figure 3e. Viral accumulation in upper uninoculated leaves of infected plants, detected using anti‐TVMV CP immunoblotting of upper uninoculated leaves, showed that inoculating *NbDCL2*, *NbDCL3* and *NbDCL4* plants with TVMV58, TVMV58mutA and TVMV58mutB after silencing resulted in virus levels similar to those in non‐silenced plants inoculated with the same viruses (Figure 5f–h). After silencing *NbDCL2*, *NbDCL3* and *NbDCL4* simultaneously in *N. benthamiana* plants (Figure 5d), TVMV58 and TVMV58 mutants were inoculated (Figure 5e), and there was no significant difference in virus accumulation compared to the control (Figure 5i). This suggested that the infectivity of TVMV58 and TVMV58 mutants is independent of the DCL homologues in *N. benthamiana*.

### 2.6 Cross‐Protection of TVMV58 Against PVY‐Ros1, TuMV‐GFP and TMV‐GFP in *N. benthamiana*

**FIGURE 6:**
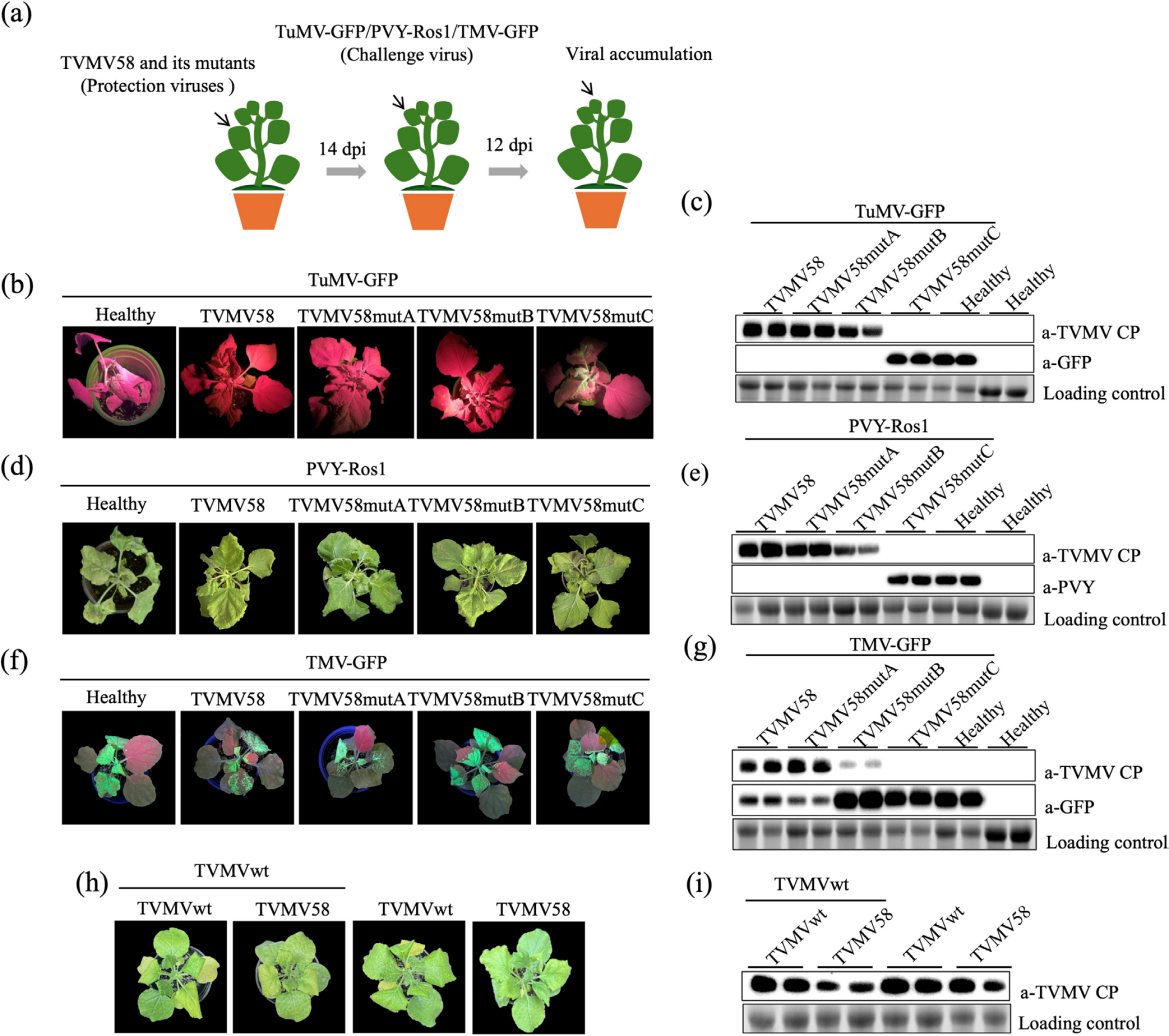
Cross‐protection of viral infection clone and mutants against PVY‐Ros1, TuMV‐GFP and TMV‐GFP in *Nicotiana benthamiana*. (a) The procedure of cross‐protection experiment is shown. The infectious clones of TVMV58. TVMV58mutA and TVMV58mutB were infiltrated in *N. benthamiana* plants. After 14 days, the upper leaves were challenge inoculated with TuMV‐GFP, PVY‐Ros1 or TMV‐GFP. (b) The viral symptoms of TuMV‐GFP in the cross‐protection assay were photographed at 7 days post‐inoculation (dpi). (c) Viral accumulation of TuMV in upper leaves were detected by western blot anti‐GFP. The accumulation of TVMV CP is shown as the control. Bands of RuBisCO are shown as a loading control. (d) The viral symptoms of PVY‐Ros1 in the cross‐protection assay were photographed at 12 dpi. (e) Viral accumulation in upper leaves were detected by western blot for CP of PVY at 12 dpi. (f) The viral symptoms of TMV‐GFP in the cross‐protection assay were photographed at 7 dpi. (e) Viral accumulation in upper leaves were detected by western blot anti‐GFP at 7 dpi. The accumulation of TVMV CP is shown as the control. Bands of RuBisCO are shown as a loading control.

Cross‐protection is defined as the phenomenon in which plants systemically infected with one virus are protected from subsequent infection by a challenge virus. To address whether the engineered viral infectious clone of TVMV58 could provide cross‐protection against potyviruses (PVY‐Ros1 and TuMV‐GFP) and tobamovirus (TMV‐GFP), these viruses were inoculated onto the leaves of plants previously infected with TVMV58 (Figure 6a). After 7 or 12 days, upper systemic leaves were collected and viral accumulation was examined.

Plants infected with TVMV58, TVMV58mutA and TVMV58mutB showed 100% protection against PVY‐Ros1 (0/36 from three experiments) and TuMV‐GFP (0/36 from three experiments) in *N. benthamiana* (Table 1), and the plants showed no viral symptoms (Figure 6b,d). However, severe symptoms of both viruses were observed in plants infected with the challenge viruses alone and TVMV58mutC (PVY‐Ros1 or TuMV‐GFP). Immunoblotting of the upper non‐inoculated leaf samples revealed that PVY CP and the viral reporter GFP of TuMV‐GFP were not detected in plants pre‐infected with TVMV58, TVMV58mutA or TVMV58mutB (Figure 6c,e). In contrast, high levels of both viruses were observed in samples challenged with PVY‐Ros1 or TuMV‐GFP. The plants infected with TVMV58, TVMV58mutA and TVMV58mutB did not show resistance to TMV‐GFP (Table 1); the plants exhibited obvious viral symptoms (Figure 6f), and the viral reporter gene GFP of TMV‐GFP was detected (Figure 6g). We also performed the cross‐protection experiment against TVMVwt. There are no clear differences in symptoms of tested plants (Figure 6h). However, less viral accumulation was detected in TVMV58‐treated plants compared with the TVMVwt‐treated plants (Figure 6i). This indicates that TVMV58 confers cross protection against TVMVwt.

**TABLE 1:** Cross protection of TVMV58 and TVMV58 mutants against PVY‐Ros1, TuMV‐GFP and TMV‐GFP.

Protection virus	Challenge virus^a^	Infectivity^b^
TVMV58	PVY‐Ros1	0/36
TVMV58mutA	PVY‐Ros1	0/36
TVMV58mutB	PVY‐Ros1	0/36
TVMV58mutC	PVY‐Ros1	36/36
Healthy	PVY‐Ros1	36/36
Healthy	—	—
TVMV58	TuMV‐GFP	0/36
TVMV58mutA	TuMV‐GFP	0/36
TVMV58mutB	TuMV‐GFP	0/36
TVMV58mutC	TuMV‐GFP	36/36
Healthy	TuMV‐GFP	36/36
Healthy	—	—
TVMV58	TMV‐GFP	36/36
TVMV58mutA	TMV‐GFP	36/36
TVMV58mutB	TMV‐GFP	36/36
TVMV58mutC	TMV‐GFP	36/36
Healthy	TMV‐GFP	36/36
Healthy	—	—

^a^ TVMV58 or TVMV58 mutants were infiltrated in *Nicotiana benthamiana*. After 14 days, upper leaves were inoculated with challenge viruses PVY‐Ros1, TuMV‐GFP or TMV‐GFP. Healthy plants were used as the control.

^b^ Total number of plants in three independent experiments that developed virus symptoms (PVY‐Ros1, TuMV‐GFP or TMV‐GFP)/total number of plants inoculated.

## 3 Discussion

Previous studies reported that the TVMV pXBS7 variant TVMVpXBS8 has a SL structure composed of 58‐nt ‘AU’ repeats in the 3′‐UTR, which caused attenuated symptoms when inoculated by its transcripts (Rodríguez‐Cerezo et al. 1991). However, the viral accumulation was similar in the mutant strains TVMVpXBS8 and TVMVwt. Zhao et al. (2020) had created the infectious clone of TVMVwt, which facilitates the study of viral infection. In this study, this 58‐nt sequence was cloned into the pLX‐TVMVwt (an infectious clone of TVMVwt) to create the mutant virus pLX‐TVMVpXBS8 (TVMV58), which allows moving the viral sequence of TVMVpXBS8 to a binary vector. Then, the plasmids were transformed into *A. tumefaciens* C58C1 and infiltrated into *N. benthamiana*. As expected, attenuated symptoms similar to those in TVMV58 were detected in TVMV58‐infected plants and without altering viral accumulation (Figures 1 and 2). Importantly, our data further confirmed that the attenuated symptoms are not associated with viral accumulation.

In recent years, the function of viral noncoding RNA in regulating host–pathogen interactions and symptom production has gradually become a hot topic in the field of virology. In 1985, a study on human viruses found that changes in the secondary structure of viral noncoding RNA can lead to changes in infection symptoms. A single base mutation in the 5′‐UTR of poliovirus RNA causes the attenuated viral pathogenicity phenotype, which is related to the secondary structure changes in this region (Bienkowska‐Szewczyk and Ehrenfeld 1988; Mathews et al. 1999).

It has been reported that the 3′ hairpin structure sequence of TSWV ambisense S RNA‐encoded mRNAs constitutes a translation enhancer that mediates efficient translation in the absence of a poly(A) tail (Geerts‐Dimitriadou et al. 2012). This is also supported by the evidence that 3′‐UTRs in plant viruses are involved in viral RNA translation. In TSWV, the S‐RNA‐derived mRNA transcription termination signal is located near the 3′ end of the intergenic hairpin structure, which can synergistically enhance cap‐dependent translation (Yang et al. 2022). To investigate the role of tobacco vein banding mosaic virus (TVBMV) 3′‐UTR in viral systemic infection, three types of deletions were introduced into the TVBMV infectious clone. Mutants with deletions at the nucleotide positions 8–42, 43–141 or 163–174 in the 3′‐UTR failed to cause systemic infection in *N. benthamiana* plants. Nucleotides at the position 8–42 near the 5′‐terminus of TVBMV 3′‐UTR could form an SL‐like structure that was crucial for TVBMV systemic movement in tobacco. We proposed that this SL‐like structure, and thus the 3′‐UTR, has an essential role in TVBMV systemic infection (Wang et al. 2019).

In this study, we designed two mutant infectious clones that break the secondary structure, resulting in TVMV58mutA with three loops and TVMV58mutB with two loops, to test whether the stem structure in the 3′‐UTR of TVMV58 was associated with the attenuated symptoms and virus accumulation. The phenotypes of *N. benthamiana* infected with TVMV58mutA and TVMV58mutB were similar to those of TVMV58; however, the amount of virus accumulated changed. This indicated that the secondary structure is essential for symptom development and may affect virus accumulation, as supported by the significantly decreased levels of viral accumulation in TVMV58mutB and slightly higher levels in TVMV58mutA at 9 dpi. We further investigated whether certain nucleotides in TVMV58mutA and TVMV58mutB were associated with attenuated symptoms and virus accumulation (Figure 3). We designed the secondary structure recapitulation mutant TVMV58mutC, which differs from TVMV58 by eight nucleotides. This mutant infectious clone construct bears a 2‐nt difference, ‘TG’, compared to TVMVwt. We found no viral infection in TVMV58mutC‐treated plants. These results suggested that the ‘UG’ in the 3′‐UTR of TVMVwt might be essential for viral infection. Therefore, we designed an infectious clone ‘TVMV‐mutTG’. These results indicated that TVMVmutTG could not infect plants. This suggested that two key nucleotide ‘UG’ sites in the 3′‐UTR of TVMV are important for viral infection (Figure 4).

In plants, RNA silencing plays a direct role in the host defence against viruses by targeting viral RNA for degradation (Pumplin and Voinnet 2013; Fukudome and Fukuhara 2017; Cordero, Cerdán, et al. 2017; Ho et al. 2007; Garcia‐Ruiz et al. 2010). DCLs are the first proteins to recognize and cut the invading viral RNA during the interaction between the virus and the gene silencing system (Ding and Voinnet 2007). Guo and colleagues in China found that DCL4 can generate siRNAs by recognizing the secondary structure of cucumber mosaic virus (CMV) satRNAs (Du et al. 2007; Duan et al. 2008; Zhu et al. 2011). Many studies have shown that DCL plays an important role in plant antiviral activity. DCL2 and DCL3 are the most relevant for host defence against potato spindle tuber viroid (PSTVd), whereas DCL4 antagonizes the function of DCL2/DCL3 (Katsarou et al. 2016). Our results showed that the specific secondary structure of TVMV58mutB with two loops affected viral infection. This may be due to the cleavage of DCLs in *N. benthamiana*. However, when *NbDCL2*, *NbDCL3* and *NbDCL4* were silenced, there was no difference in the symptoms or viral accumulation in plants infected with TVMV58, TVMV58mutA and TVMV58mutB. This indicated that the decreased accumulation of TVMV58, TVMV58mutA and TVMV58mutB was independent of NbDCLs (Figure 5).

Cross‐protection against several viruses has been successfully applied to prevent crop yield losses at the field scale (Ziebell and MacDiarmid 2017). For example, zucchini yellow mosaic virus (ZYMV), papaya ringspot virus (PRSV), cocoa swollen shoot virus (CSSV), citrus tristeza virus (CTV) and PepMV have been used to identify mild strains and their ability to protect against the most severe strains and have been successfully applied to crops (Ziebell and Carr 2010; Folimonova 2013; Ameyaw et al. 2016; Lee and Keremane 2013). Based on this approved cross‐protection method, attention should be paid to a broader range of plant viruses that are available for use in cross‐protection. After infiltration with TVMV58 or its mutant viral infectious clone in healthy *N. benthamiana*, challenge inoculation with TuMV‐GFP, PVY‐Ros1 and TMV‐GFP was performed on upper leaves at 14 dpi. TVMV58, TVMV58mutA and TVMV58mutB, but not TVMV58mutC, displayed cross‐protection against PVY‐Ros1 and TuMV‐GFP. For TMV‐GFP, TVMV58, TVMV58mutA and TVMV58mutB did not show resistance (Figure 6). This indicates that TVMV58 and its mutant viruses only exhibit cross protection against viruses of the same genus and do not have cross protection against viruses of other genera.

In summary, this is a novel report that the 3′‐UTR in TVMV is indispensable in viral symptoms and viral infections, especially as its secondary structure plays a significant role. Moreover, silencing of *DCL2*/*3*/*4* does not affect TVMVwt and TVMV58 infection. This indicates that the 3′‐UTR of TVMVwt and TVMV58 is not processed by the cleavage of DCLs, which in turn facilitates engineering of the TVMV 3′‐UTR for cross‐protection against further viral infections. Our findings revealed key functions of the 3′‐UTR in plant viruses, providing insight into the general mechanism of viral pathogenicity and informing the development of 3′‐UTR‐based disease management strategies.

## 4 Experimental Procedures

### 4.1 Plant Materials

Wild‐type *N. benthamiana* plants were sown directly on soil and grown in a greenhouse (light/dark cycle of 16/8 h at 22°C–24°C).

### 4.2 Viruses and Viral Propagation

PVY‐Ros1, a PVY isolate carrying the *Rosea1* gene, was donated by José‐Antonio Daròs, Instituto de Biología Molecular y Celular de Plantas (Cordero, Mohamed, et al. 2017). TuMV‐GFP was obtained from Xiaoming Zhang's laboratory at the Institute of Zoology at the Chinese Academy of Sciences. TMV‐GFP was previously described (Casper and Holt 1996).

PVY‐Ros1, TuMV and TMV‐GFP were propagated in *N. benthamiana* plants. The systemic leaves with typical infection symptoms were collected and stored at −80°C for viral infection assay. Viral inoculation with PVY‐Ros1 and TuMV was performed as previously described (Pasin et al. 2020).

### 4.3 Agroinfiltration of the Virus in *N. benthamiana*

*Agrobacterium tumefaciens* C58C1 cells carrying infectious clones were grown to OD_600_ = 0.5. A disposable syringe (1 mL) without a needle was used to infiltrate the abaxial side of the leaves, avoiding the vein, so that the bacterial solution could spread throughout the leaves. After infiltration, the inoculated plants were placed in a greenhouse. The symptoms were observed and documented using photography.

### 4.4 Construction of Infectious Clones of TVMV58 and Mutants

The TVMV infectious clone pLX‐TVMVwt (TVMVwt) was created using a one‐step Gibson assembly with a custom enzymatic premix (Zhao et al. 2020). An infectious clone of pLX‐TVMVwt was used as the wild‐type virus and backbone to construct the recombinant virus. We synthesized a fragment including the 58‐nt 'AT' fragment and cloned it into the pMK vector to create plasmid pMK‐TVMV58 (Figure S1a). The sequencing data showed that the insertion fragment contained partial sequences of TVMV CP, TVMV 3′‐UTR, 58 nt 'AT' and pLXB vector. Primer sets 1F/1R and 2F/2R were used to amplify the two fragments (Table S1). Two fragments spanning the TVMV genome and pLX backbone, including the 58‐nt 'AT'‐rich fragment, were assembled with a homemade enzyme mix to create pLX‐TVMV58 (TVMV58) (Figure S1b). PCR amplification of the 58‐nt ‘AT’ stable segment in TVMV58 (1#–5#) plants was performed using primers mut‐F/mut‐R (Figure S2a), followed by Sanger sequencing (Figure S2b).

The 275 bp fragments were amplified from pMK‐mutA and pMK‐mutB using the primers mut‐F/mut‐R (Figure S3a). A specific fragment (13,458 bp) was obtained using PCR amplification using primers TVMV58‐F/TVMV58‐R and the template plasmid pLXB‐TVMVpXBS8 (Figure S3b). The PCR amplification products were recombined using one‐step Gibson assembly ligation to obtain pLX‐TVMV58mutA (TVMV58mutA) and pLX‐TVMV58mutB (TVMV58mutB) (Zhao et al. 2020). PCR fragments of 275 bp were amplified from TVMV58mutA and TVMV58mutB to confirm the positive clones (Figure S3c). Sanger sequencing verification results after plasmid extraction showed that mutant nucleotides were introduced into both TVMV58mutA and TVMV58mutB plasmids (Figure S3d,e). The TVMV58, TVMV58mutA and TVMV58mutB infectious clone plasmids were transformed into *A. tumefaciens* C58C1. A 968 bp fragment was obtained using PCR amplification with 2F/2R primers from *A. tumefaciens* C58C1 carrying the plasmids TVMV58mutA and TVMV58mutB (Figure S4).

The 275 bp fragment was amplified from the pMK‐mutC plasmid using the primers mut‐F/mutC‐R (Figure S5a). A 13490 bp fragment was obtained from the pLXB‐TVMV58mutB plasmid using PCR amplification using the primers TVMV58mutC‐F/TVMV58mutC‐R (Figure S5b). The two fragments were recombined using one‐step Gibson A assembly ligation to create a viral infectious clone, pLX‐TVMV58mutC (TVMV58mutC). The 275 bp fragment was amplified from the plasmid TVMV58mutC by PCR with mut‐F/mut‐R primers to identify the correct clone (Figure S5c). Sanger sequencing verification results after plasmid extraction showed that the mutant nucleotides were introduced into the TVMV58mutC plasmid (Figure S5d). This confirmed that the correct infectious clone for the TVMV58mutC mutant was obtained.

The primers TVMVpoint‐F/TVMVpoint‐R were designed to introduce the ‘T/G’ mutation at positions 10,428 nt and 10,436 nt in the 3′‐UTR of pLX‐TVMVwt. A 13,648 bp fragment was amplified from the pLX‐TVMVwt plasmid using the primers TVMVpoint‐F/TVMVpoint‐R (Figure S6a). The linearized fragment was treated with DpnI, phosphorylated, self‐ligated to form a circular closed plasmid and transformed into *Escherichia coli* DH5α. The plasmid was identified by PCR using 2F/2R primers, and a 910 bp fragment was obtained (Figure S6b). The plasmid was verified by XbaI digestion, and two fragments of 2277 bp and 11,318 bp were obtained (Figure S6c). The Sanger sequencing proved that the TVMVmutTG infectious clone contained the ‘T/G’ nucleotide mutation (Figure S6d). The TVMVwt, TVMV58mutC and TVMVmutTG clones were transformed into *A. tumefaciens* C58C1. The PCR fragments were amplified from *A. tumefaciens* C58C1 carrying the plasmids TVMV58mutC (968 bp), TVMV58mutTG (910 bp) and TVMVwt (910 bp) (Figure S7).

### 4.5 Construction of *NbDCL* Silencing Vectors

Fragments of *DCL2* (195 bp), *DCL3* (173 bp) and *DCL4* (200 bp) were obtained using PCR amplification from *N. benthamiana* plants (Figure S8a). The *NbDCL2*, *NbDCL3* and *NbDCL4* fragments were digested with XbaI/XhoI, and the fragments were ligated into the pTRV2 vector (Figure S8b) using T4 DNA ligase. The ligation reaction solution was transformed into *E. coli* DH5α. Positive transformants were verified using PCR (Figure S8c,d). Sanger sequencing indicated that the TRV‐mediated silencing vectors pTRV2‐NbDCL2, pTRV2‐NbDCL3 and pTRV2‐NbDCL4 had been successfully constructed. The plasmids were transformed into *A. tumefaciens* C58C1 cells. Primers pTRV1/pTRV2 were used to amplify the fragments of corresponding sizes from the *Agrobacterium* carrying the plasmids to identify the right colonies (Figure S8e).

### 4.6 Determination of Virus Accumulation Using Western Blot Analysis

Vial accumulation in the tested plants was detected 14 dpi (Zhao et al. 2020). For each treatment, the upper systemic leaves from eight plants were collected and the virus accumulation was determined using western blotting with anti‐TVMV CP immunoblotting. The CP signal was quantified using ImageJ software. An independent samples *t*‐test was performed to determine the significance of the differences (*p* < 0.05).

### 4.7 Virion Observation

Virion observations in virus‐infected samples were performed as previously described (Zhao et al. 2020). Briefly, plant extracts were prepared in 5 mM Tris–HCl (pH 7.5), 150 mM NaCl and 2.5 mM dithiothreitol (DTT), and incubated with collodion‐coated carbon‐stabilized copper grids percolated with the anti‐TVMV CP serum. The grids were negatively stained with 2% uranyl acetate and observed under a transmission electron microscope (JEM 1011, Jeol), and images were taken with an ES1000W Erlangshen CCD camera (Gatan). ImageJ software was used for image processing.

### 4.8 Passage Inoculation of TVMV58 to Verify the Infection Stability

The recombinant virus TVMV58 was agro‐infiltrated into the leaves of *N. benthamiana*. The upper systemic leaves were collected at 14 dpi and used as an inoculum for the next passage of infection. Each infection from the 2th to the 5th passages was conducted using manual inoculation of *N. benthamiana* plants. The symptoms of passage‐infected plants were observed and photographed 14 dpi.

### 4.9 Cross‐Protection Assays

TVMV58 was used as a protective agent in cross‐protection assays. TuMV‐GFP, PVY‐Ros1 and TMV‐GFP were used as challenge viruses. The 2.5‐week‐old *N. benthamiana* seedlings were infiltrated with *A. tumefaciens* C58C1 containing the TVMV58 or its mutant viruses infectious clones. The upper leaves were mechanically inoculated with the challenge viruses PVY‐Ros1, TuMV‐GFP or RMV‐GFP, which was prepared from 0.5 g of infected *N. benthamiana* leaves using 1 mL of 0.01 M sodium phosphate buffer (pH 7.2), 14 days after inoculation with the protective virus. Protection was evaluated by examining the development of symptoms in the plants. The presence of the challenge virus was detected using western blotting analysis with anti‐CP antibodies for PVY‐Ros1 or anti‐GFP antibodies for TuMV‐GFP and TMV‐GFP.

### 4.10 Reverse Transcription‐Quantitative PCR

Total RNA was extracted using a TRIzol isolation kit (HLINGENE) according to the manufacturer's protocol. Complementary DNA (cDNA) was synthesized with dT_18_ using an Evo M‐MLV RT Mix Kit with gDNA Clean for qPCR Ver. 2 (Accurate Biology). Quantitative PCR was performed in a 20 μL volume system containing 10 μL of 2× SYBR Green Pro Taq HS Premix (ROX plus), 1 μL of 10‐fold diluted cDNA, 0.4 μL of 10 μM of each primer and RNA‐free water. Amplification was performed using QuantStudio 3 and 5 Real‐Time PCR Systems MAN0010407 (Thermo Fisher Scientific). Expression was normalized to that of the *N. benthamiana ACTIN* gene (GenBank accession no. AY179605) as an internal control, and the 2^−ΔΔ*C*t^ method was used to calculate the level of gene expression (Wang et al. 2025).

### 4.11 Protein Structure Prediction

RNAFold was used to predict the secondary structures of TVMVwt, TVMV58, TVMV58mutA, TVMV58mutB and TVMV58mutC using default settings. The structures were visualized and profiled on a website (http://rna.tbi.univie.ac.at/cgi‐bin/RNAWebSuite/RNAfold.cgi).

### 4.12 Statistical Analysis

The signals of the CP bands were quantified using the ImageJ software. Significant differences were analysed using the GraphPad Prism software.

## Author Contributions

**Zhenqi Sun:** software, data curation, methodology. **Zhaoran Wu:** software, data curation, visualization. **Jiaxin Xu:** software, data curation, methodology, visualization. **Baolong Zhang:** software, data curation, visualization. **Mingmin Zhao:** conceptualization, methodology, software, data curation, funding acquisition, writing – original draft, writing – review and editing. **Haijuan Wang:** software, data curation, visualization, conceptualization, writing – original draft, writing – review and editing, methodology.

## Funding

This work was supported by National Natural Science Foundation of China (31860489).

## Conflicts of Interest

The authors declare no conflicts of interest.

## Supporting information


**Figure S1:** The pMK‐TVMV58 plasmid map and synthetic sequences. (a) The map of plasmid pMK‐TVMV58 and synthetic sequences. The 5′ black uppercase base is a partial CP sequence of TVMVwt. The partial sequence of TVMVwt 3′ noncoding RNA shown in blue. The repeat sequence, containing 58 nt and rich in ‘AU’ is shown in red. The black lowercase base is a partial sequence of pLX vector from pLX‐TVMVwt. (b) Sequencing map of the 58 nt AT sequence in the pMK‐TVMV58 plasmid, highlighted with a blue background.

**Figure S2:** Validation of the stability of TVMV58 (1#–5#).(a) Amplification of TVMV58 (1#–5#) insertion fragments (275 bp). M: Marker (1 kb plus DNA Ladder). (b) PCR product Sanger sequencing.

**Figure S3:** Construction and sequencing of infectious clones TVMV58mutA and TVMV58mutB. (a) Amplification of Pmk‐mutA and pMK‐mutB insertion fragments (275 bp). M: Marker (1 kb plus DNA Ladder). (b) Amplification of TVMV58‐2 vector fragment (13,458 bp). M: Marker (D15000 + 2000 DNA Ladder). (c) PCR fragments of 275 bp amplified from TVMV58mutA and TVMV58mutB to confirm the positive clones. M: Marker (1 kb and DNA Ladder). (d) Sequencing map of TVMV58mutA indicating mutant nucleotides (red square). (e) Sequencing map of TVMV58mutB indicating mutant nucleotides (red square).

**Figure S4:** PCR fragment (968 bp) amplified from *Agrobacterium tumefaciens* C58C1 carrying plasmids TVMV58mutA and TVMV58mutB.

**Figure S5:** Construction and sequencing of TVMV58mutC infectious clone. (a) Amplification of pMK‐mutC insertion fragments (275 bp). M: Marker (1 kb plus DNA Ladder). (b) Amplification of TVMV58‐3 vector fragment (13,490 bp). M: Marker (D15000 + 2000 DNA Ladder). (c) PCR fragments of 275 bp amplified from TVMV58mutC to confirm the positive clones. M: Marker (1 kb plus DNA Ladder). (d) Sequencing map of TVMV58mutC to indicate the mutant nucleotides (red square).

**Figure S6:** Construction and sequencing of TVMV58mutTG infectious clone. (a) Amplification of TVMVmutTG‐1 vector fragment (13,648 bp). M: Marker (1 kb plus DNA Ladder). (b) Amplification of pMK‐mutTG insertion fragments (910 bp). M: Marker (1 kb plus DNA Ladder). (c) Enzyme digestion of TVMVmutTG by XbaI resulted in 11,318 bp and 2277 bp. M: Marker (1 kb plus DNA Ladder). (d) Sequencing map of TVMV58mutTG to indicate the mutant nucleotides (red square).

**Figure S7:** PCR fragments amplified from *Agrobacterium tumefaciens* C58C1 carrying plasmids TVMV58mutC, TVMV58mutTG and TVMVwt.

**Figure S8:** Construction of *NbDCL1*, *NbDCL2*, *NbDCL3* and *NbDCL4* gene silencing vectors. Amplification of fragments of *NbDCL2* (173 bp), *NbDCL3* (195 bp), and *NbDCL4* (200 bp). M: Marker (DL500 plus DNA Ladder). (b) pTRV2 plasmids were digested using EcoRI/XhoI and XbaI/XhoI, which resulted in 9627 bp and 9721 bp fragments. M: Marker (1 kb plus DNA Ladder). (c) PCR fragments of 453 bp amplified from pTRV2‐NbDCL3 to confirm the positive clone. M: Marker (DL500 plus DNA Ladder). (d) PCR fragments of 453 bp amplified from pTRV2‐NbDCL2 and pTRV2‐NbDCL4 to confirm the positive clones. M: Marker (1 kb plus DNA Ladder). (e) PCR fragments amplified from *Agrobacterium tumefaciens* bacterial solution carrying pTRV2‐NbDCL2, pTRV2‐NbDCL3, pTRV2‐NbDCL4, pTRV1 and pTRV2. M: Marker (1 kb plus DNA Ladder).

**Table S1:** Primer sequences.

## Data Availability

The data that support the findings of this study are available from the corresponding author upon reasonable request.
