# Supplementary figures and images for: Engineering the 3′‐UTR of Tobacco Vein Mottling Virus to Confer Cross‐Protection Against Potyviruses
Source: Mol Plant Pathol. 2026 May 1;27(5):e70268. doi: 10.1111/mpp.70268 (PMC13135073; doi:10.1111/mpp.70268)

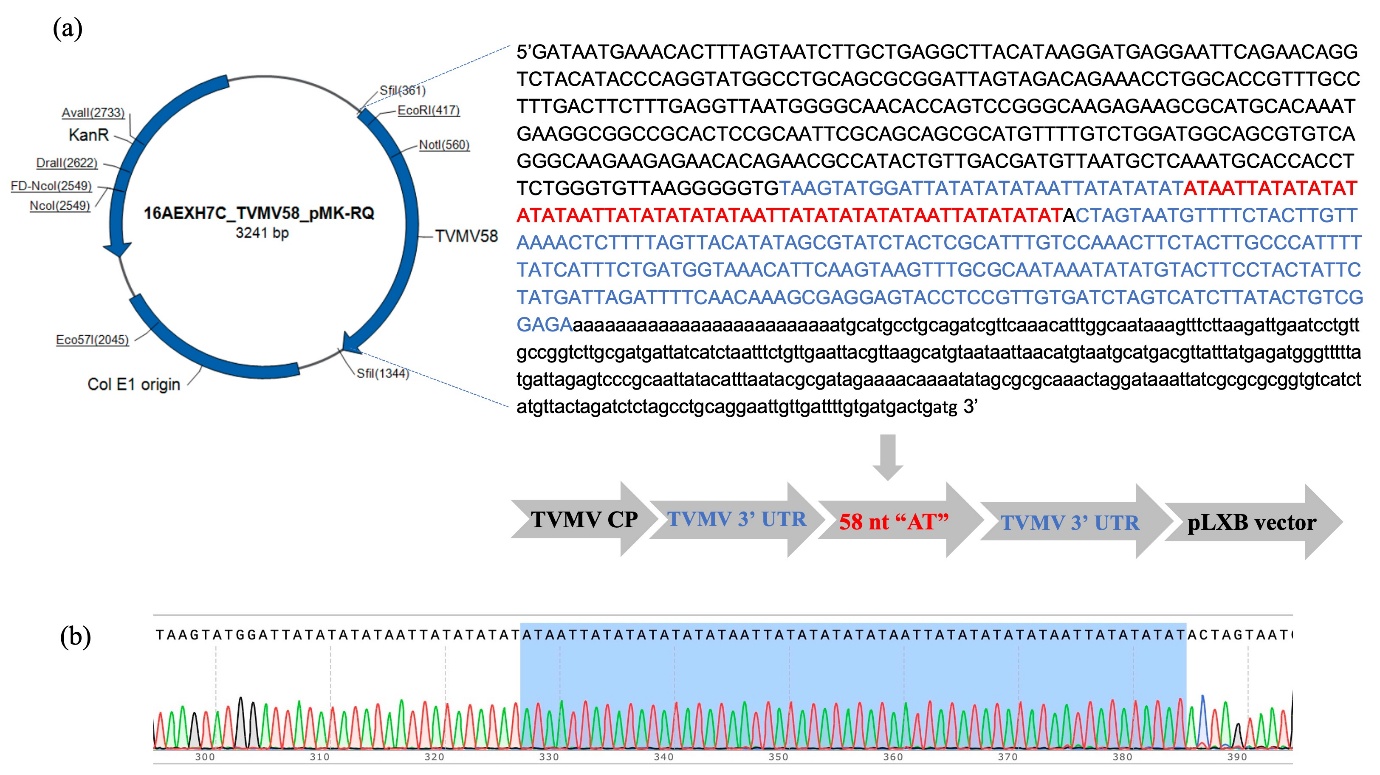


Figure S1

Supplement: Supplementary file 1 — Figure S1: The pMK‐TVMV58 plasmid map and synthetic sequences. (a) The map of plasmid pMK‐TVMV58 and synthetic sequences. The 5′ black uppercase base is a partial CP sequence of TVMVwt. The partial sequence of TVMVwt 3′ noncoding RNA shown in blue. The repeat sequence, containing 58 nt and rich in ‘AU’ is shown in red. The black lowercase base is a partial sequence of pLX vector from pLX‐TVMVwt. (b) Sequencing map of the 58 nt AT sequence in the pMK‐TVMV58 plasmid, highlighted with a blue background. [file MPP-27-e70268-s008.docx]

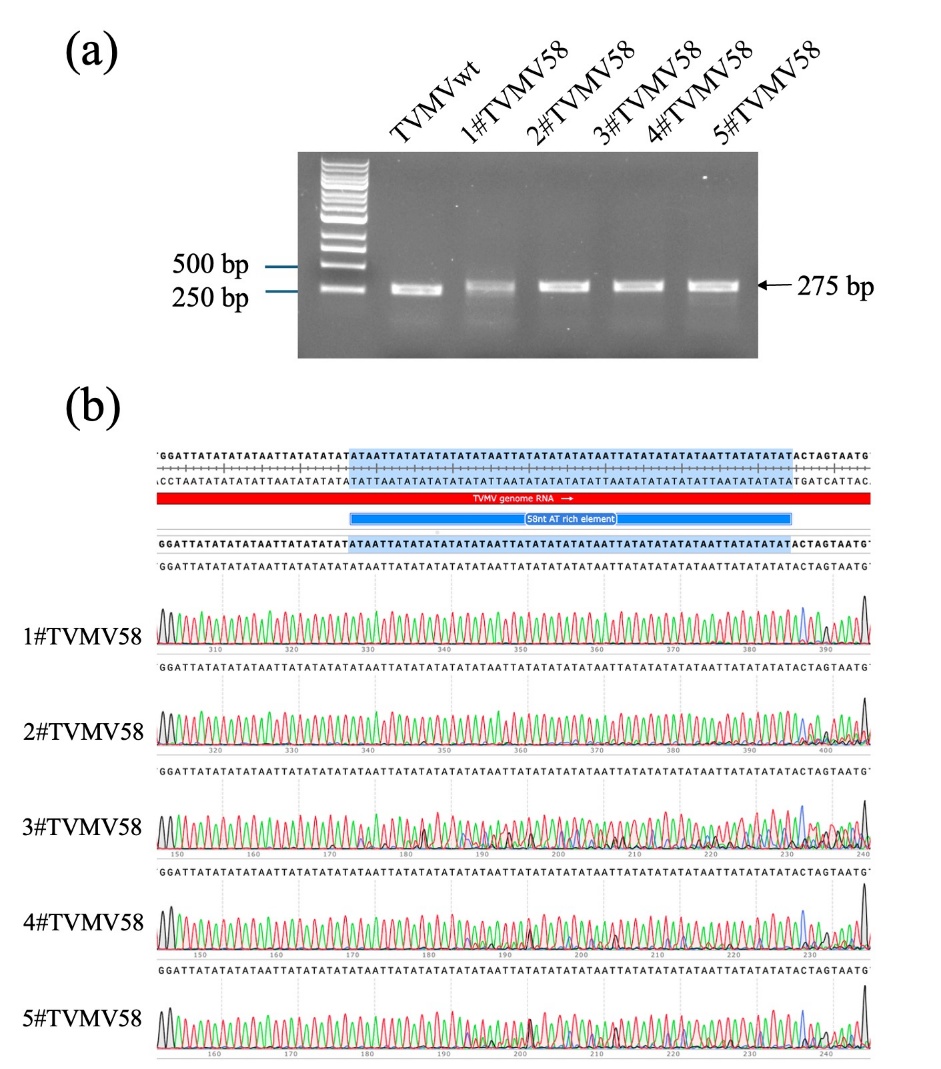


Figure S2

Supplement: Supplementary file 2 — Figure S2: Validation of the stability of TVMV58 (1#–5#).(a) Amplification of TVMV58 (1#–5#) insertion fragments (275 bp). M: Marker (1 kb plus DNA Ladder). (b) PCR product Sanger sequencing. [file MPP-27-e70268-s009.docx]

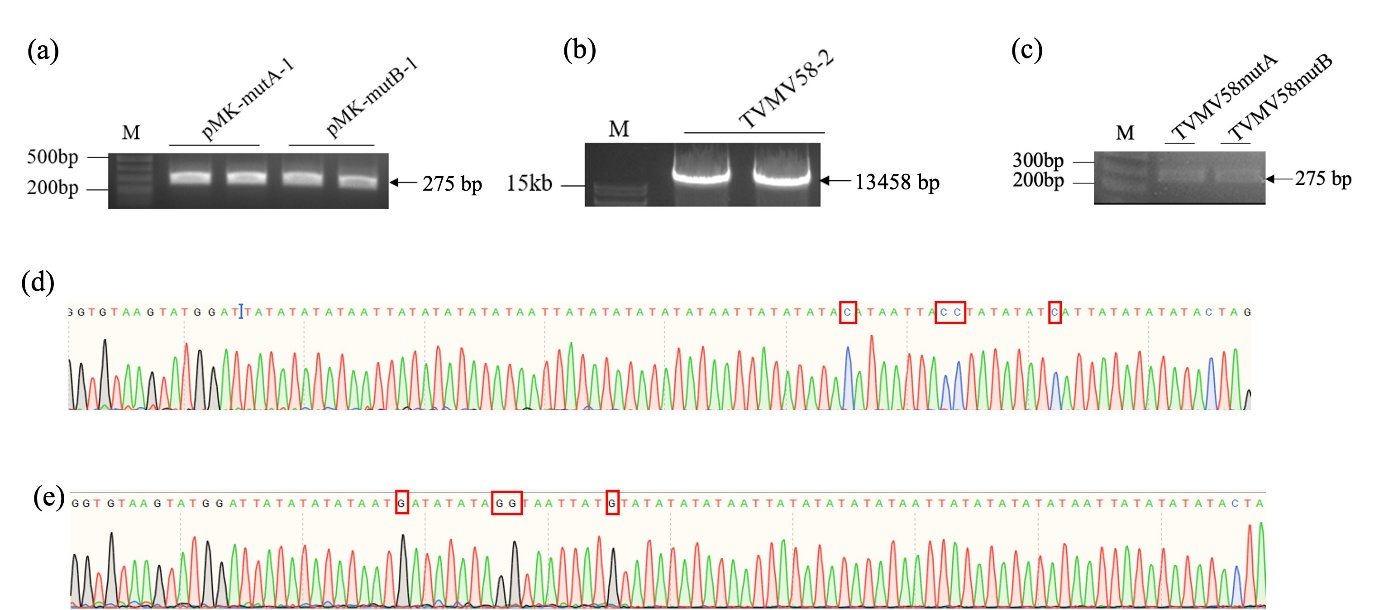


Figure S3

Supplement: Supplementary file 3 — Figure S3: Construction and sequencing of infectious clones TVMV58mutA and TVMV58mutB. (a) Amplification of Pmk‐mutA and pMK‐mutB insertion fragments (275 bp). M: Marker (1 kb plus DNA Ladder). (b) Amplification of TVMV58‐2 vector fragment (13,458 bp). M: Marker (D15000 + 2000 DNA Ladder). (c) PCR fragments of 275 bp amplified from TVMV58mutA and TVMV58mutB to confirm the positive clones. M: Marker (1 kb and DNA Ladder). (d) Sequencing map of TVMV58mutA indicating mutant nucleotides (red square). (e) Sequencing map of TVMV58mutB indicating mutant nucleotides (red square). [file MPP-27-e70268-s007.docx]

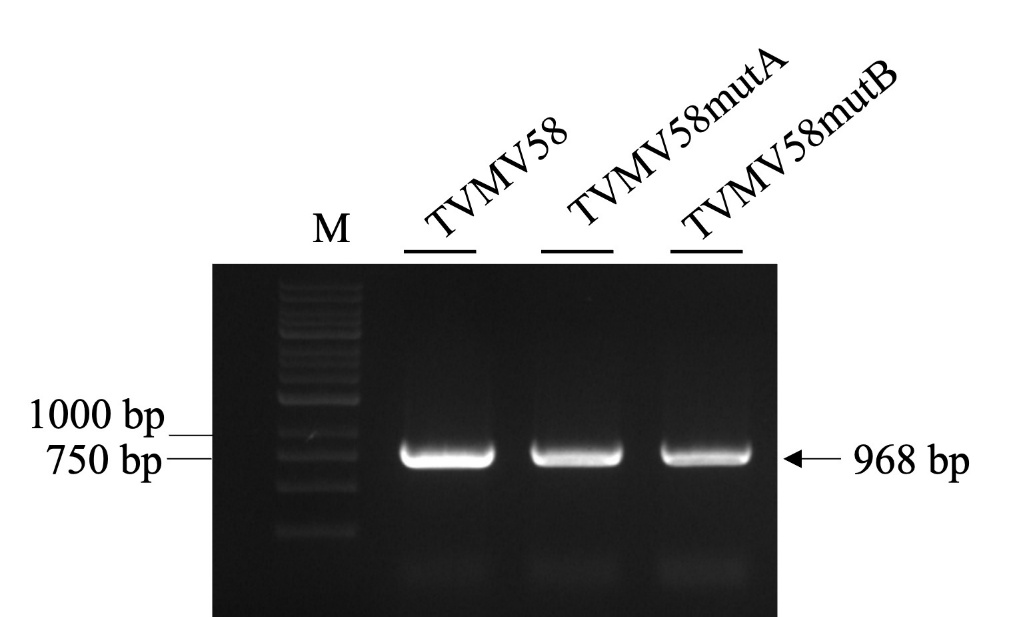


Figure S4

Supplement: Supplementary file 4 — Figure S4: PCR fragment (968 bp) amplified from Agrobacterium tumefaciens C58C1 carrying plasmids TVMV58mutA and TVMV58mutB. [file MPP-27-e70268-s003.docx]

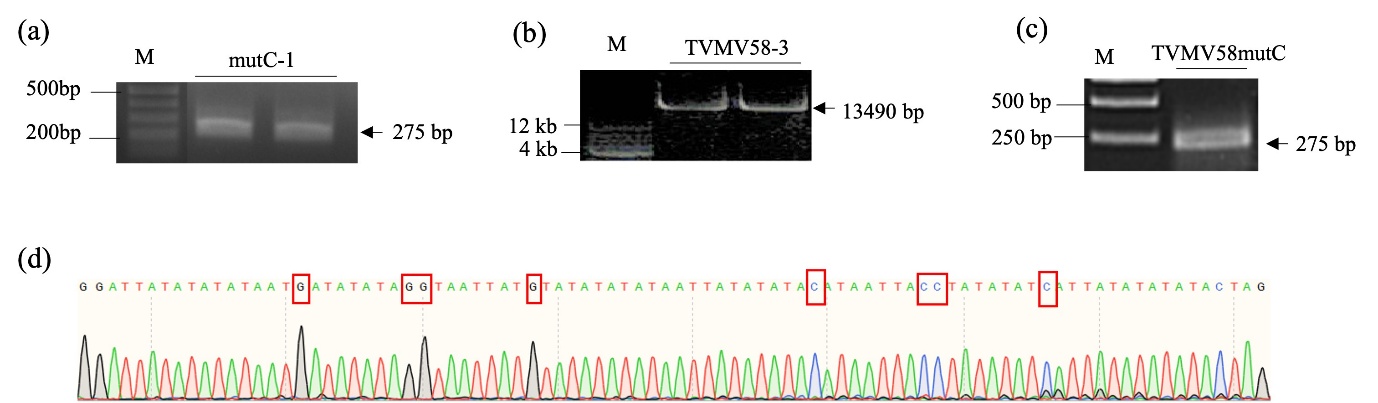


Figure S5

Supplement: Supplementary file 5 — Figure S5: Construction and sequencing of TVMV58mutC infectious clone. (a) Amplification of pMK‐mutC insertion fragments (275 bp). M: Marker (1 kb plus DNA Ladder). (b) Amplification of TVMV58‐3 vector fragment (13,490 bp). M: Marker (D15000 + 2000 DNA Ladder). (c) PCR fragments of 275 bp amplified from TVMV58mutC to confirm the positive clones. M: Marker (1 kb plus DNA Ladder). (d) Sequencing map of TVMV58mutC to indicate the mutant nucleotides (red square). [file MPP-27-e70268-s004.docx]

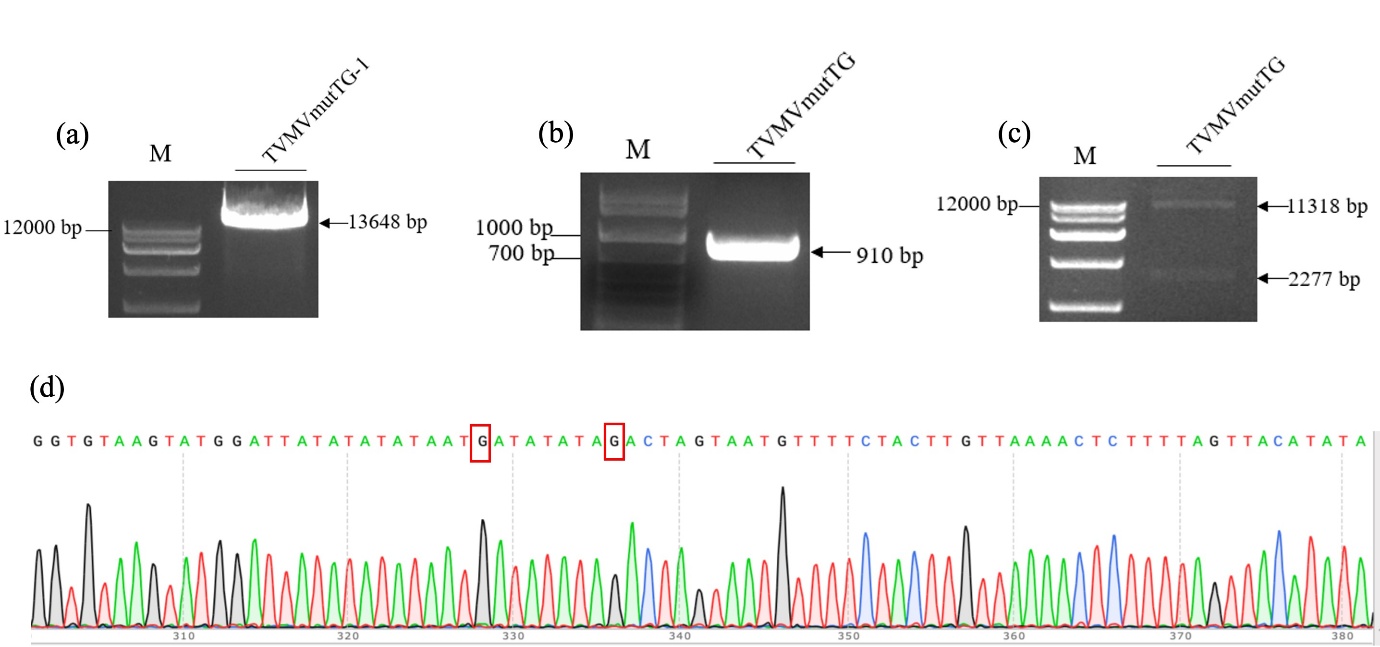


Figure S6

Supplement: Supplementary file 6 — Figure S6: Construction and sequencing of TVMV58mutTG infectious clone. (a) Amplification of TVMVmutTG‐1 vector fragment (13,648 bp). M: Marker (1 kb plus DNA Ladder). (b) Amplification of pMK‐mutTG insertion fragments (910 bp). M: Marker (1 kb plus DNA Ladder). (c) Enzyme digestion of TVMVmutTG by XbaI resulted in 11,318 bp and 2277 bp. M: Marker (1 kb plus DNA Ladder). (d) Sequencing map of TVMV58mutTG to indicate the mutant nucleotides (red square). [file MPP-27-e70268-s005.docx]

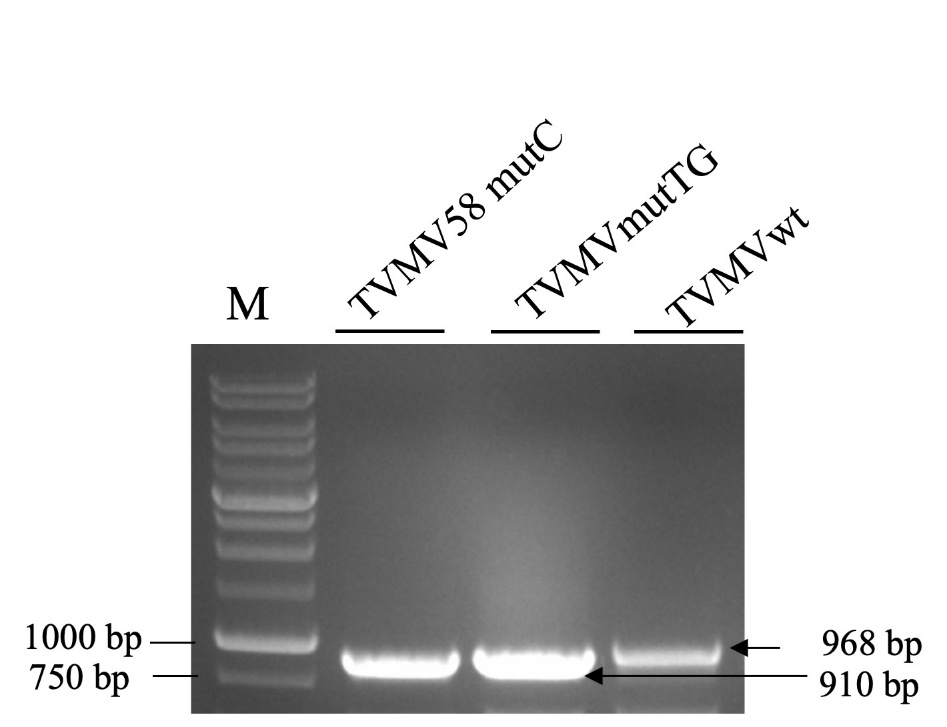


Figure S7

Supplement: Supplementary file 7 — Figure S7: PCR fragments amplified from Agrobacterium tumefaciens C58C1 carrying plasmids TVMV58mutC, TVMV58mutTG and TVMVwt. [file MPP-27-e70268-s006.docx]

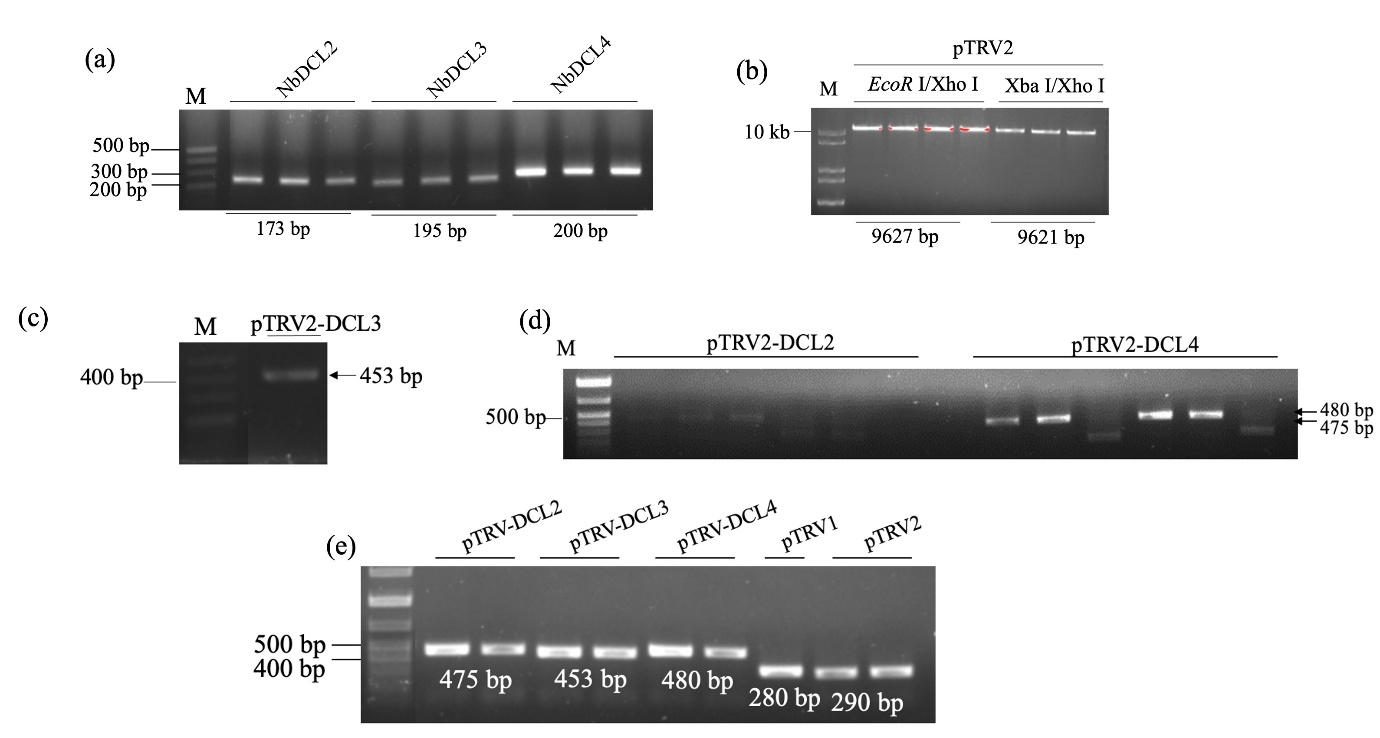


Figure S8

Supplement: Supplementary file 8 — Figure S8: Construction of NbDCL1, NbDCL2, NbDCL3 and NbDCL4 gene silencing vectors. Amplification of fragments of NbDCL2 (173 bp), NbDCL3 (195 bp), and NbDCL4 (200 bp). M: Marker (DL500 plus DNA Ladder). (b) pTRV2 plasmids were digested using EcoRI/XhoI and XbaI/XhoI, which resulted in 9627 bp and 9721 bp fragments. M: Marker (1 kb plus DNA Ladder). (c) PCR fragments of 453 bp amplified from pTRV2‐NbDCL3 to confirm the positive clone. M: Marker (DL500 plus DNA Ladder). (d) PCR fragments of 453 bp amplified from pTRV2‐NbDCL2 and pTRV2‐NbDCL4 to confirm the positive clones. M: Marker (1 kb plus DNA Ladder). (e) PCR fragments amplified from Agrobacterium tumefaciens bacterial solution carrying pTRV2‐NbDCL2, pTRV2‐NbDCL3, pTRV2‐NbDCL4, pTRV1 and pTRV2. M: Marker (1 kb plus DNA Ladder). [file MPP-27-e70268-s002.docx]
