# Supplementary material for: Engineering the 3′‐UTR of Tobacco Vein Mottling Virus to Confer Cross‐Protection Against Potyviruses
Source: Mol Plant Pathol. 2026 May 1;27(5):e70268. doi: 10.1111/mpp.70268 (PMC13135073; doi:10.1111/mpp.70268)
Supplement: Supplementary file 9 — Table S1: Primer sequences. [file MPP-27-e70268-s001.docx]

Table S1 Primer sequences

| Primer | Primer sequence （ 5’-3’ ） |
| --- | --- |
| 1 F | GGAATTGTTGATTTTGTGATGACTG |
| 1 R | CAAGATTACTAAAGTGTTTCATTATC |
| 2 F | GATAATGAAACACTTTAGTAATCTT |
| 2 R | CATCAGTCATCACAAAATCAACAATTCC |
| mut-F | AGAAGCGCATGCACAAATGAAGGCG |
| mut-R | TTAACAAGTAGAAAACATTACTAGT |
| TVMV58-F | TATACTAGTAATGTTTTCTACTTGTTAA |
| TVMV58-R | GGCCGCCTTCATTTGTGCATGCGCTTCT |
| mut-F | AGAAGCGCATGCACAAATGAAGGCG |
| mutC-R | TTAACAAGTAGAAAACATTACTAGTATATATATAATGATATATAGGTAATTATGTATATATAA |
| TVMV58mutC-F | TTATATATACATAATTACCTATATATCATTATATATATACTAGTAATGTTTTCTACTTGTTAA |
| TVMV58mutC-R | CGCCTTCATTTGTGCATGCGCTTCT |
| TVMVpoint-F | TATATATAATgATATATAgACTAGTAATGTTTTCTACTTGTTAAAACTCTT |
| TVMVpoint-R | CATTACTAGTcTATATATcATTATATATATAATCCATACTTA |
| pTRV2-DCL2-F | TGCTCTAGATTATGAAATGGCTTGGGATC |
| pTRV2-DCL2-R | CCGCTCGAGGATAGCATCGTGGAATCTCA |
| pTRV2-DCL3-F | TGCTCTAGATGTTGAATGCGGTGAAGGCT |
| pTRV2-DCL3-R | CCGCTCGAGGTCGTTCTAGCTCATACAGC |
| pTRV2-DCL4-F | TGCTCTAGACGTCCGTGCCCAGAAATCTG |
| pTRV2-DCL4-R | CCGCTCGAGAATGCAATTGCCGCTTTGAA |
| qPCR- DCL2_F | GCTTGGGATCGACATTGATT |
| qPCR- DCL2_R | ATTCCAAGCGCTGATAGCAT |
| qPCR- DCL3_F | GTGGCCTATGCCTAGTTTGG |
| qPCR- DCL3_R | TCAGCTCTTTCATCCCCAGT |
| qPCR- DCL4_F | AGCGGCAATTGCATTTCTTA |
| qPCR- DCL4_R | CATCTATTTCATCAGCAAGTGGA |
| ACTIN_F | TGGTCGTACCACCGGTATTGTGTT |
| ACTIN_R | TCACTTGCCCATCAGGAAGCTCAT |
| TRV1_F | AGTATTTCCACACAGGAGAAAGAAC |
| TRV1_R | GGTCTAGTAGTAGCTGGTCT |
| TRV2_F | GTTCAGGCGGTTCTTGTGTG |
| TRV1_R | CCGATCAATCAAGATCAGTCG |
